# A comprehensive analysis of somatic alterations in Chinese ovarian cancer patients

**DOI:** 10.1038/s41598-020-79694-0

**Published:** 2021-01-11

**Authors:** Yingli Zhang, Xiaoliang Shi, Jiejie Zhang, Xi Chen, Peng Zhang, Angen Liu, Tao Zhu

**Affiliations:** 1 Department of Gynecologic Oncology, Institute of Cancer and Basic Medicine (ICBM), Chinese Academy of Science, Hangzhou, People’s Republic of China; 2 Department of Gynecological Surgery, Cancer Hospital of the University of Chinese Academy of Sciences, Hangzhou, People’s Republic of China; 3 Department of Gynecological Surgery, Zhejiang Cancer Hospital, No 1, East Banshan Road, Gongshu District, Hangzhou, 310022 People’s Republic of China; 4 OrigiMed Co. Ltd, Shanghai, 201114 People’s Republic of China

**Keywords:** Cancer genomics, Tumour biomarkers, Biomarkers, Molecular medicine, Cancer

## Abstract

Ovarian cancer is one of the most common cancers in women and is often diagnosed as advanced stage because of the subtle symptoms of early ovarian cancer. To identify the somatic alterations and new biomarkers for the diagnosis and targeted therapy of Chinese ovarian cancer patients, a total of 65 Chinese ovarian cancer patients were enrolled for detection of genomic alterations. The most commonly mutated genes in ovarian cancers were *TP53* (86.15%, 56/65), *NF1* (13.85%, 9/65), *NOTCH3* (10.77%, 7/65), and *TERT* (10.77%, 7/65). Statistical analysis showed that *TP53* and *LRP1B* mutations were associated with the age of patients, *KRAS*, *TP53*, and *PTEN* mutations were significantly associated with tumor differentiation, and *MED12*, *LRP2*, *PIK3R2*, *CCNE1*, and *LRP1B* mutations were significantly associated with high tumor mutational burden. The mutation frequencies of *LRP2* and *NTRK3* in metastatic ovarian cancers were higher than those in primary tumors, but the difference was not significant (*P* = 0.072, for both). Molecular characteristics of three patients responding to olapanib supported that *BRCA* mutation and HRD related mutations is the target of olaparib in platinum sensitive patients. In conclusion we identified the somatic alterations and suggested a group of potential biomarkers for Chinese ovarian cancer patients. Our study provided a basis for further exploration of diagnosis and molecular targeted therapy for Chinese ovarian cancer patients.

## Introduction

Ovarian cancer is one of the most common gynecological malignant tumors worldwide. Since morbidity and mortality are increasing year by year, ovarian cancer has become a real threat to women's health and survival^1,2^. Patients with early stage ovarian cancer can typically undergo surgical treatment, but those with advanced stage or metastasis usually do not have specific treatment options, and therefore, the prognosis is poor^3,4^. The initial treatment for advanced ovarian cancer is usually debulking surgery followed by adjuvant chemotherapy or neoadjuvant therapy^5^. However, 5-year survival rates of ovarian cancer are less than 50%^2^. Therefore, understanding the mechanism of tumorigenesis and developing potential biomarkers for diagnosis and targeted therapy would be of great clinical value for ovarian cancer.

Next-generation sequencing (NGS) technology has been widely used in molecular genetic research of various tumors^6–8^. Recently, a series of studies on the mutational landscape of ovarian cancer have been reported^9,10^. Balendran et al. identified the most commonly altered genes in brain metastatic ovarian cancer to be *BRCA1/2*, *TP53*, and *ATM*^10^. *TP53* was the most commonly mutated gene in all ovarian cancer subtypes^9^. A study on a cohort of patients with epithelial ovarian cancer identified 77.4% of patients having at least one somatic or germline mutation, and that the most frequent germline and somatic mutations were *BRAC1/2* and *TP53* mutations, respectively^11^. There are many subtypes of ovarian cancer and these histological types have different carcinogenesis mechanisms, which leads to potentially different treatment options for each subtype^12,13^. According to the FIGO Cancer Report 2018, epithelial ovarian cancers were the most common type, and high-grade serous ovarian cancer (HGSOC) was the most common subtype^14^. Garziera et al. found a heterogeneous mutational landscape and poorer prognosis in HGSOC patients harboring concurrent mutations of 2 driver actionable genes in the 26-cancer-gene panel^15^. Targeted NGS showed potential advantage for identifying subgroups of patients with distinct therapeutic vulnerabilities based on the mutational profile expressed by ovarian cancers^15^. A bioinformatics analysis of mutational and clinical data of 334 HGSOC tumor samples from The Cancer Genome Atlas (TCGA) revealed a sub-cluster of high-frequency mutations in 58 genes associated with DNA damage repair, apoptosis, and cell cycle regulation in 22 patients, indicating that germline mutations of *CHECK2*, *RPS6KA2*, and *MLL4* genes could be used as a risk factor predictor for women^16^. Also, Kuo et al. had reported that *CDKN2A/B*, *CSMD1*, and *DOCK4* were the most common alterations in HGSOC^17^, and Ganapathi et al. found that *COL2A1* and pseudogene *SLC6A10P* could be used for predicting tumor recurrence in HGSOC^18^. However, patients from different races or geographic regions often have different mutational characteristics. Analyzing the mutational characteristics of patients in China could complement and improve the knowledge of molecular characteristics of ovarian cancers as a whole and provide evidence for diagnosis and targeted therapy for ovarian cancers. Therefore, we enrolled 65 Chinese ovarian cancer patients in this study and performed NGS testing to identify characteristics of genomic alterations (GAs) and potential biomarkers for diagnosis and targeted therapy of ovarian cancers.

## Patients and methods

### Patient enrollment and sample collection

A total of 65 ovarian cancer patients were enrolled in this study from Zhejiang Cancer Hospital. Informed consent was obtained from all patients and this study was approved by the Institutional Ethics Committee. Both formalin-fixed, paraffin-embedded (FFPE) tumor tissues, including 49 primary lesions, 10 metastatic lesions, and 6 lesions with unknown origin, and matched blood samples were collected from enrolled patients. FFPE samples containing at least 20% of tumor cells were used for NGS detection. Genomic DNA was isolated by using QIAamp DNA FFPE Tissue Kit and QIAamp DNA Blood Midi Kit (Qiagen, Hilden, Germany) according to the manufacturer’s instructions. The concentration of DNA was measured by Qubit and normalized to 20–50 ng/μL.

### Identification of GAs and tumor mutational burden (TMB)

The genomic information of 59 ovarian cancer patients was produced by using the YuanSu450 gene panel, which covers all of the coding exons of 450 cancer-related genes and 64 selected introns in 39 genes that are frequently rearranged in solid tumors (supplementary material 1), and the genomic information of 6 olaparib sensitive patients was produced by whole exome sequencing (WES). The mean depth of Yuansu450 gene panel was 800 × (range: 320–2727), and the mean depth of WES was 500 × (range: 122–1814). All sequencing data were obtainedby using Illumina NextSeq 500 (Illumina, Inc., CA) in OrigiMed laboratory certified by College of American Pathologists (CAP) and Clinical Laboratory Improvement Amendments (CLIA). GAs were identified followed previous study^19,20^. Single nucleotide variants (SNVs) were identified by MuTect (v1.7). Insertion-deletions (Indels) were identified by using PINDEL (V0.2.5). The raw calls of SNV and short Indel were further selected as follows: A minimum of 5 reads was required to support alternative calling. Variants with read depths less than 30 × with strand bias larger than 10% or VAF < 0.5% were removed. The functional impact of GAs was annotated by SnpEff3.0. Copy number variation (CNV) regions were identified by Control-FREEC (v9.7) with the following parameters: window = 50,000 and step = 10,000. Gene fusions/rearrangements were detected through an in-house developed pipeline: paired-end reads with abnormal insert size of over 2000 bp aligned to the same chromosome or aligned to different chromosomes were collected and a discordant paired clusters according to the pairing relationship, then consistent breakpoints from the paired-end discordant reads within a cluster were identified to establish potential fusion/rearrangement breakpoints. Gene fusions/rearrangements were assessed by Integrative Genomics Viewer (IGV). Germline variants were filtered from database of the 1000 Genomes. TMB was calculated by counting the somatic mutations, including SNVs and Indels, per megabase of the sequence examined in each patient. The variation information can be found in supplementary materials 2.

### Statistical analysis

Statistical analyses were performed by using SPSS version 22.0 (SPSS Inc., Chicago, IL, USA). Fisher’s exact test was used to analyze significant differences. Bonferroni correction were performed for multiple test correction. *P* < 0.05 was considered statistically significant.

### Ethical statement

The project was approved by the Ethic Committee of Zhejiang Cancer Hospital. We declare that all methods used in this protocol were carried out in accordance with relevant guidelines and regulations. This study was approved by all patients and all participants provided informed consent.

## Results

### Clinical characteristics of ovarian cancer patients

A total of 65 Chinese ovarian cancer patients with a median age of 54 years (range 33–84 years) were enrolled in this study. These samples consisted of 50 (76.92%) high-grade serous adenocarcinomas, 6 (9.23%) low-grade serous adenocarcinomas, 1 (1.54%) mucinous adenocarcinoma, 1 (1.54%) clear cell tumor, 1 (1.54%) endometrioid tumor, and 6 (9.23%) unknown tumor subtypes. Forty-nine (75.38%) samples were primary lesions, and 10 (15.38%) samples were metastatic lesions. The lesion sites of 6 (9.23%) samples were unknown. According to TNM staging system, which considered the tumor size (T), lymph node/lymph node diffusion (N), and tumor metastasis (M), 5 (7.69%) patients were in stage I, 8 (12.31%) patients were in stage II, , 33 (50.77%) patients were in stage III, 9 (13.8%) patients were in stage IV, and 8 (12.31%) patients with unclear tumor stage. Histologically, 4 (6.15%) tumors were well/moderately differentiated, and 53 (81.54%) tumors were poorly/undifferentiated ovarian cancer. The differentiation information of 8 (12.31%) tumors was unknown. Patients’ clinical or pathological information was summarized and shown in Table 1.

**Table 1:** Clinicopathologic features of 65 ovarian cancer patients.

Total	65	
Age	Median (range)	54 (33–84)
TMB	Median (range)	4.1 (0.6–23.2)
Tumor site	Primary lesion	49 (75.38%)
	Metastatic lesion	10 (15.38%)
	Unknown	6 (9.23%)
Histology	High-grade serous adenocarcinoma	50 (76.92%)
	Low-grade serous adenocarcinoma	6 (9.23%)
	Mucinous adenocarcinoma	1 (1.54%)
	Clear cell tumor	1 (1.54%)
	Endometrioid tumor	1 (1.54%)
	Unknown	6 (9.23%)
Tumor stage	Stage I	5 (7.69%)
	Stage II	8 (12.31%)
	Stage III	35 (53.85%)
	Stage IV	9 (13.85%)
	Unknown	8 (12.31%)
Tumor differentiation	Well/moderately differentiated	4 (6.15%)
	Poorly/undifferentiated	53 (81.54%)
	Unknown	8 (12.31%)

### GAs in ovarian cancer

**Figure 1:**
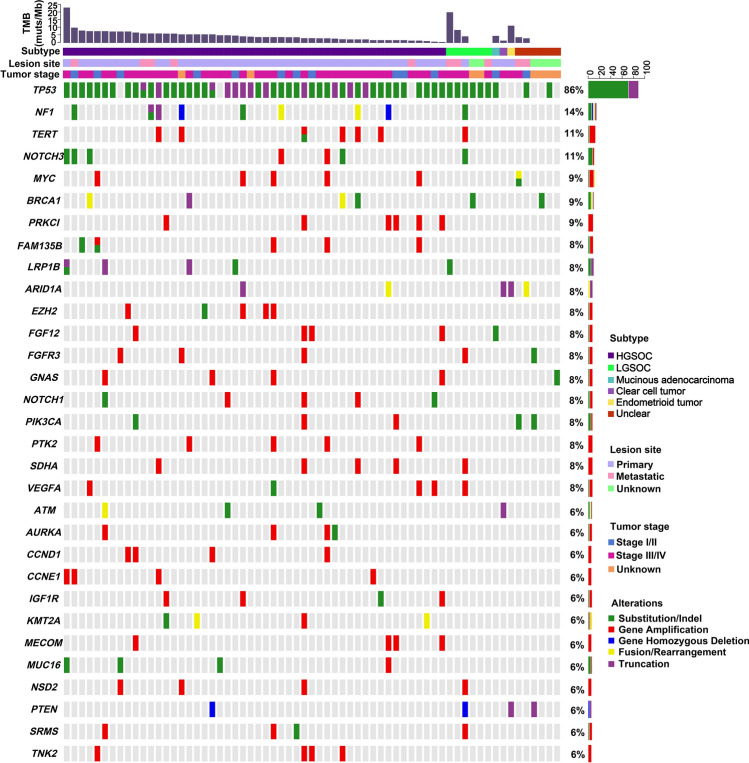
Mutational landscape of 65 ovarian cancer patients. The X-axis represents each case sample and the Y-axis represents each mutated gene. The bar graph above shows the tumor mutational burden (TMB) value of each sample, and the bar graph on the right shows the mutation frequency of each mutated gene in 65 samples. Green represents substitution/Indel mutations, red represents gene amplification mutations, blue represents gene homozygous deletion mutations, yellow represents fusion/rearrangement mutations, and purple represents truncation mutations.

A total of 595 clinically relevant somatic GAs in 317 genes were identified by using NGS sequencing targeting 450 cancer genes and WES, with a mean of 9.15 alterations per sample (range 1–29). GAs included 285 (47.90%) SNV/ShortIndels, 263 (44.20%) CNVs, 31 (5.21%) fusions, and 16 (2.69%) LongIndels (Table S1). The most commonly mutated genes were *TP53* (86.15%, 56/65), *NF1* (13.85%, 9/65), *NOTCH3* (10.77%, 7/65), and *TERT* (10.77%, 7/65) (Fig. 1). Notably, most *TP53* mutations were SNVs and mainly occurred in the DNA-binding domain. In this cohort, 39 mutation sites of *TP53* were detected, with the most common mutation being R273H, which was detected in 5 cases, followed by Y220C in 4 cases, and R248Q/W in 4 cases (Figure S1). Most *TP53* mutations were detected once in this cohort. Although 7 GAs of *TERT* were detected, most of them were CNVs (Fig. 1). Germline mutations were detected in 25 of 65 OC patients, including mutations from 17 *BRCA1*, 3 *BRCA2*, 3 *RAD51D*, 1 *RAD51C*, and 1 *FANCA*. Except for 4 *BRCA1* and 1 *BRCA2* mutations which belong to SNV, the others are truncation (Table S2).

### Mutation characteristics of ovarian cancer patients at different ages

Patients were divided into 3 groups based on age: 32–49 years old (27.69%, 18/65), 50–59 years old (38.46%, 25/65), and 60–84 years old (33.85%, 22/65). In patients aged 32–49 years, 180 GAs from 145 genes were detected (10 GAs/patient and 8.1 genes/patient), and the most commonly mutated genes included *TP53*, *FGFR3*, *MYC*, and *NSD2*. In patients aged 50–59 years, 224 GAs from 162 genes were detected (8.96 GAs/patient and 6.48 genes/patient), and the most commonly mutated genes included *TP53*, *NF1*, *BRAC1*, and *NOTCH3*. In patients aged 60–84 years, 156 GAs from 112 genes were detected (7.1 GAs genes/patient and 5.1 genes/patient), and the most commonly mutated genes included *TP53*, *LRP1B*, *CCNE1*, *LRP2*, and *NOTCH1* (Table S3).

**Figure 2:**
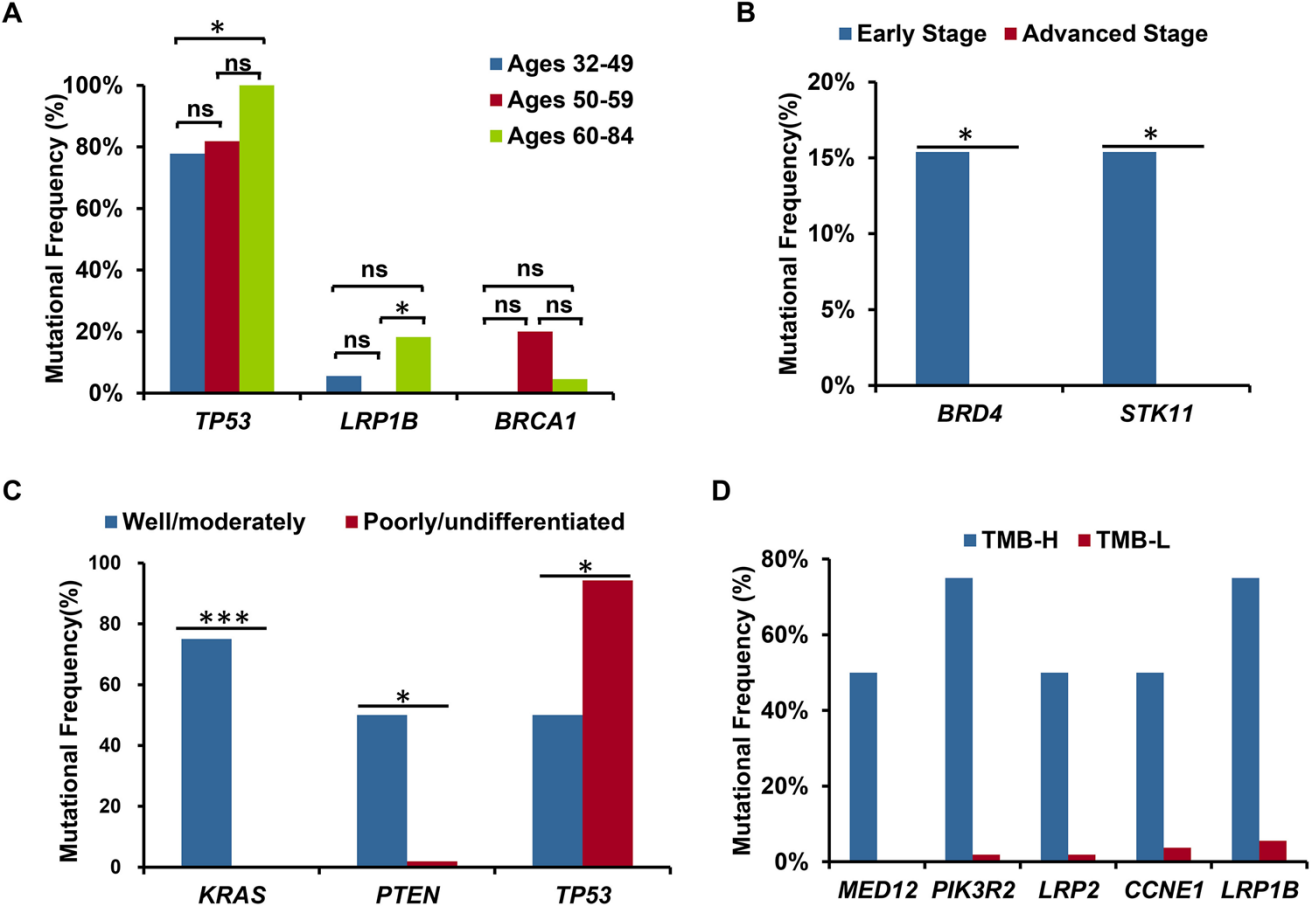
Correlated analysis of mutated genes and clinical characteristics. (**A**) Correlation analysis of mutated genes and the age of patients; (**B**) Correlation analysis of mutated genes and tumor stage; (**C**) Correlation analysis of mutated genes and tumor differentiation; and (**D**) Correlation analysis of mutated genes and TMB. The X-axis shows the mutated genes and the Y-axis represent the mutational frequency of each gene. Fisher’s exact test was used to analyze significant differences and bonferroni correction were performed for multiple test correction. ns *P* > 0.05, * P < 0.05, ** P < 0.01, and *** P < 0.001.

Statistical analysis showed that the frequencies of *TP53* and *LRP1B* mutations were significantly higher in patients aged 60–84 years compared to that in the other 2 age groups (*P* = 0.02 and *P* = 0.04, respectively), while the frequency of *BRAC1* mutations was significantly higher in patients aged 50–59 years compared to the other 2 age groups (*P* = 0.03). Multiple comparison showed that *TP53* mutation was significantly more frequent in 60–84 years group than that in 32–49 years group, and *LRP1B* mutation was significantly more frequent in 60–84 years group than that in 50–59 years group (Fig. 2A). Among the mutated genes with more than 2 GAs in this cohort, *FGFR2* and *FLI1* mutations specifically occurred in patients aged 32–49 years, *FGFR1, CREBBP, NFKBIA,* and *RUNX1* mutations specifically occurred in patients aged 50–59 years, and *LRP2, CHD4, CRLF2, EPHA3, KDM6A, KMT2C* mutations specifically occurred in patients aged 60–84 years (Table S3). Although the differences of mutation frequency in each patient group were not significant, these specific gene mutations might be potential biomarkers that correlate with the age of ovarian cancer patients.

### The mutation of *BRD4 and STK11* is associated with the tumor stage of ovarian cancer

Patients were divided into 2 groups based on tumor stages: patients with stage I and stage II tumors (13 patients) and patients with stage III and stage IV (44 patients). Interestingly, we found that *BRD4* and *STK11* mutations were specifically detected in patients with stage I and II tumors. Statistical analysis showed that the frequency of *BRD4* and *STK11* mutations were significantly higher in stage I/II tumors than that in stage III/IV tumors (15.4% vs. 0%, *P* = 0.049, for both) (Fig. 2B).

### The correlation analysis between mutated genes and tumor differentiation of ovarian cancer

Based on the tumor differentiation of ovarian cancer, we divided patients into 2 groups, patients with well/moderately differentiated tumors (4 patients) and patients with poorly/undifferentiated tumors (53 patients). In the 4 patients with well/moderately differentiated tumors, mutations of *KRAS*, *TP53*, and *PTEN* were detected as the most frequently mutated genes. Statistical analysis showed that the mutation frequencies of *KRAS* and *PTEN* were significantly higher in well/moderately differentiated tumors than that in poorly/undifferentiated tumors, while the mutation frequency of *TP53* was significantly higher in poorly/undifferentiated tumors than that in well/moderately differentiated tumors (Fig. 2C). Although the mutation frequency of some genes, such as *NF1*, *PRKCI*, and *BRCA1*, were higher in the poorly/undifferentiated tumors than those in well/moderately differentiated tumors, the differences were not significant according to statistical analysis (Table S4).

### GAs of ovarian cancer patients in primary and metastatic lesions

Based on the tumor lesion site, samples were divided into primary lesions and metastatic lesions. The most commonly mutated genes in 49 primary lesions were *TP53*, *NF1*, *LRP2*, and *NTRK3*, while in 10 metastatic lesions, the most commonly mutated genes were *TP53*, *NF1*, *TERT*, *NOTCH3*, and *PRKCI*. The mutation frequency of *LRP2* and *NTRK3* in metastatic tumors was higher than those in primary tumors (2% vs. 20%, *P* = 0.072, for both). However, due to the small number of samples, the statistical analysis results were not significant (Table 2).

**Table 2:** Correlation analysis between mutated genes and the origin of tumor samples.

Genes	Primary lesions (49)		Metastatic lesions (10)		P-value (Fisher’s exact)
	Number of mutations	Mutational frequency (%)	Number of mutations	Mutational frequency (%)	
TP53	45	91.84	8	80.00	0.266433
LRP2	1	2.04	2	20.00	0.07152
NTRK3	1	2.04	2	20.00	0.07152
TERT	7	14.29	0	0.00	0.590097
PRKCI	6	12.24	0	0.00	0.576789
EZH2	5	10.20	0	0.00	0.576789
FAM135B	5	10.20	0	0.00	0.576789
FGF12	5	10.20	0	0.00	0.576789
NOTCH1	5	10.20	0	0.00	0.576789
PTK2	5	10.20	0	0.00	0.576789
SDHA	5	10.20	0	0.00	0.576789
VEGFA	5	10.20	0	0.00	0.576789
BRCA1	4	8.16	0	0.00	0.805958

### The correlation between gene variations and TMB

To explore the correlation between TMB and clinically relevant GAs, we measured TMB in all samples. The median TMB of 65 samples was 4.1 muts/Mb (ranges 0.6–23.2 muts/Mb) (Table 1). Four patients were identified as high TMB (TMB-H), defined as TMB values more than 10 muts/Mb, and 54 patients were identified as low TMB (TMB-L), defined as TMB values lower than 10 muts/Mb. TMB values were not identified in 7 patients. The mutations of *LRP1B*, *OBSCN*, *PIK3R2*, *TP53*, *CCNE1*, *LRP2*, *MED12*, and *NOTCH3* frequently occurred in patients with TMB-H, while the mutations of *TP53*, *NF1*, *TERT*, *MYC*, *FAM135B*, and *PRKCI* frequently occurred in patients with TMB-L (Table S5). Statistical analysis showed that the mutation frequencies of *MED12*, *LRP2*, *PIK3R2*, *CCNE1*, and *LRP1B* were significantly higher in patients with TMB-H than that in patients with TMB-L (Fig. 2D).

### The molecular characteristics of high grade serous ovarian cancer patients in China are different from those in the West

**Figure 3:**
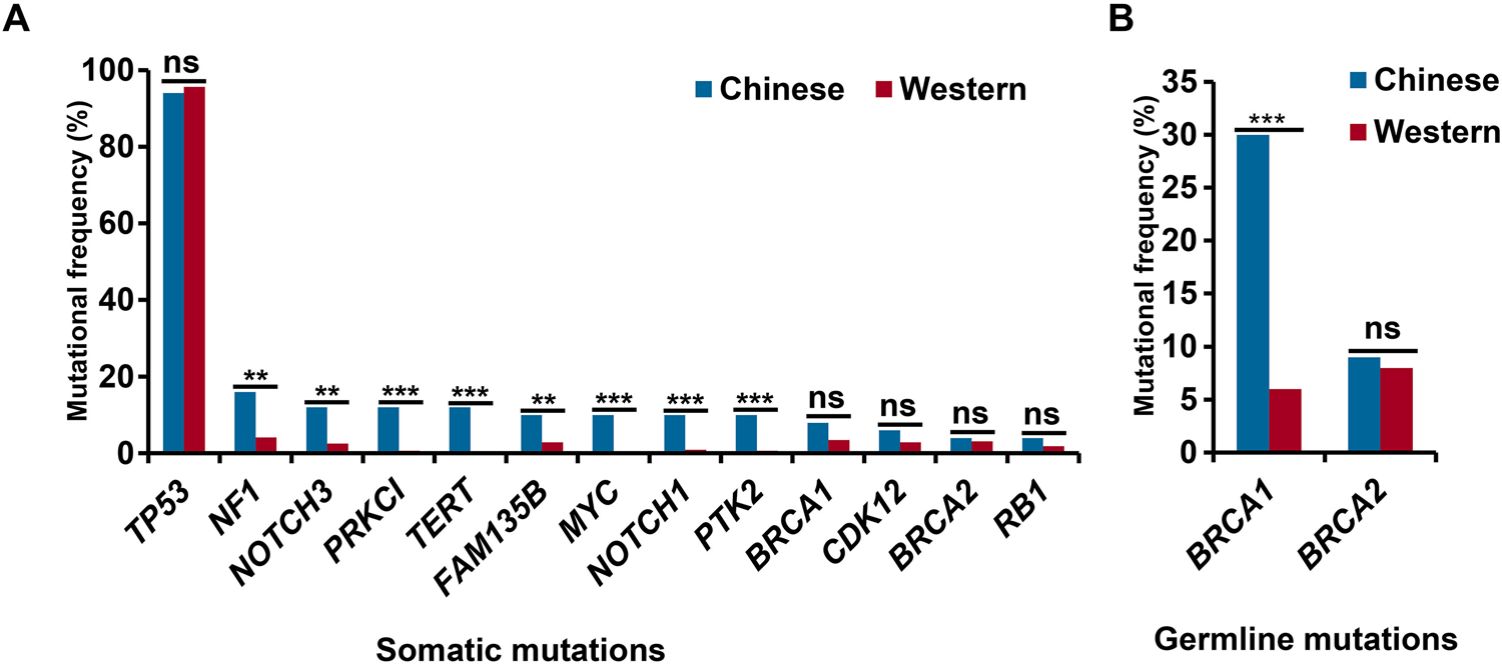
Different molecular characteristics of high-grade serous ovarian cancer patients in China and Western countries. (**A**) Differences of somatic mutations between Chinese and Western patients. (**B**) Differences of germline mutations between Chinese and Western patients. The X-axis shows the mutated genes and the Y-axis represents the mutational frequency of genes. Fisher’s exact test was used to analyze significant differences. ns *P* > 0.05, ** P < 0.01, and *** P < 0.001.

Among 65 ovarian samples, 56 were serous ovarian cancer including 50 HGSOC and 6 low-grade serous ovarian cancer. The most somatic mutations were *TP53* (94%), *NF1* (16%), *NOTCH3*, *PRKCI*, and *TERT* (12%, for each), and *FAM135B*, *MYC*, *NOTCH1*, and *PTK2* (11%, for each). According to the report from TCGA^21^, we compared the somatic mutational characterization of ovarian cancers between Chinese and Western. Statistical analysis showed that the mutational frequencies of *NF1* (*P* = 0.0024), *NOTCH3* (*P* = 0.004), *PRKCI* (4.49 × 10^–6^), *TERT* (4.44 × 10^–7^), *FAM135B* (0.008), *MYC* (4.44 × 10^–7^), *NOTCH1* (2.71 × 10^–5^), and *PTK2* (4.49 × 10^–6^) in Chinese ovarian cancer patients were significantly higher than those in Western patients (Fig. 3A). Although the mutations of *BRCA1* and *BRCA2* are the most common germline mutations in ovarian cancer, the mutational frequency of *BRCA1* is significantly higher in Chinese than in Western (*P* = 0.0001), while the mutation frequency of *BRCA2* is similar between them (*P* = 0.78) (Fig. 3B).

### Molecular characteristics of platinum sensitive patients and their response to olaparib

**Figure 4:**
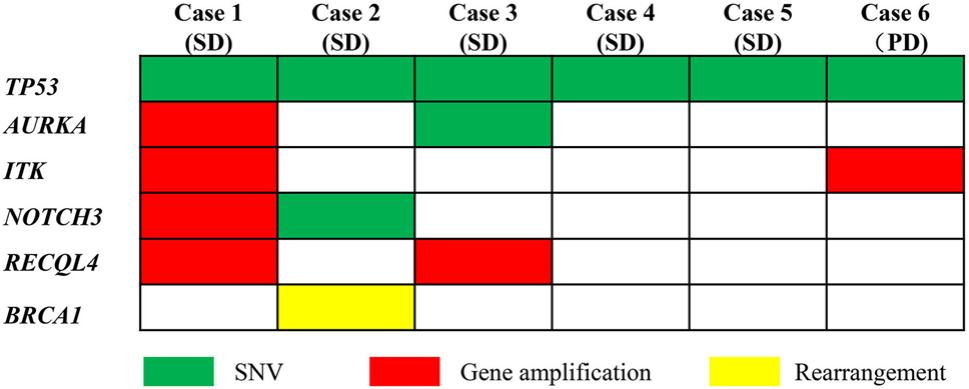
Most frequent mutated genes in 6 platinum sensitive patients. Green represents SNV mutations, red represents gene amplification mutations, and yellow represents rearrangement. SD, stable disease; PD, progressive disease.

Patients who relapsed more than 6 months after the last platinum chemotherapy are considered to be platinum sensitive. Six platinum sensitive patients were treated with olaparib at the recommended dose of 200 mg twice a day. Five of them (case 1–5) responded well and one (case 6) failed to response. To understand the molecular feature of these patients, WES was performed for further mutated gene detection. The most frequent mutated gene of 6 cases were *TP53*, *AURKA*, *ITK*, *NOTCH3*, *RECQL4*, and *BRCA1* and the other 110 mutated genes were detected only once (Fig. 4, Table S6). We found that only case 2 harbored the mutation of *BRCA1* rearrangement, the amplifications of *AURKA* and *RAD21* were detected in case 1, and *AURKA* SNV was detected in case 3. Most of mutations in case 1 were gene amplification, while most of mutations in other cases were SNV. In addition to SNV and gene amplification mutations, deletion of *PARK2*, *QKI*, *GATA4*, *PML*, *PRKAR1A*, and *LRP1B* were detected in case 6 (Table S6).

## Discussion

Ovarian cancer is a heterogeneous disease in morphology and biology. Effectively identifying gene variations is of great importance to personalized medicine and determining potential therapeutic targets for ovarian cancer. NGS technology is a potentially effective method for identifying subgroups of patients based on their genomic characteristics. Here, we identified the mutational profile of 65 ovarian cancer samples, most of which were HGSOC. Consistent with previous studies, *TP53* was also the most frequently detected gene mutation^9–11^. *TP53* is a well-known tumor suppressor gene which can regulate key transcription factors of DNA repair, apoptosis, aging, and stress metabolism^22^. The mutation of *TP53* may lead to inactivation of the p53 pathway and activation of multiple carcinogenic pathways^23^. The mutations of *TP53* can be classified as gain-of-function or loss-of-function^24,25^. Previously, Garziera et al*.* successfully identified 6 new mutation sites of *TP53* in HGSOC patients by using NGS^26^. Although 38 mutation sites of *TP53* were detected in this cohort, none of them were the same. These results indicate that *TP53* variants are complex and each mutation site that was detected might be a potential target for further therapy. A high frequency of *TP53* mutations was associated with a poor prognosis in many cancers, including HGSOC^27^. A *TP53* mutation frequency of more than 80% was detected in this cohort and may suggest a poor prognosis of ovarian cancer patients. In addition to *TP53* mutations, *KRAS*, *PIK3CA*, *PTEN*, and *BRCA1* mutations were also commonly detected in ovarian cancer^10,28^. However, a lower mutation frequency of *KRAS*, *PIK3CA*, and *BRCA1* was detected in this cohort, which may be due to most patients having HGSOC.

Patients’ age, tumor stage, and histological subtype are the most important prognostic factors^29^. Based on 104 patients with epithelial ovarian cancer, Ashour et al*.* showed that 86.4% of patients harboring *BRCA1/2* mutations were younger than 50 years old, suggesting that the age at diagnosis was a strong predictor of the presence of pathogenic *BRCA1/2* mutations^30^. In another study of stage II-IV high-grade epithelial ovarian cancer, deleterious germline *BRCA* mutations were detected and patients’ mean age at diagnosis was younger for patients harboring *BRCA1* mutations than patients harboring *BRCA2* mutations (52 years vs. 57 years, respectively, *P* = 0.06)^31^. Zhu et al*.* showed that serous ovarian cancer had a significantly higher *BRCA1* hypermethylation frequency compared to non-serous ovarian cancer, but there was no significant correlation between *BRCA1* hypermethylation and age^32^. Consistent with previous studies, we also found that *BRCA1* mutations occurred with a higher frequency in the younger age group, but there was no significant difference between each age group. Notably, a high frequency of *TP53* mutations occurred in older patients in this cohort, suggesting a worse prognosis for older patients.

*LRP1B* is a tumor suppressor that interacts with uPAR to inhibit cell migration^33^. *LRP1B* was reported to be a potential factor for chemoresistance in HGSOC patients^34^. Similar to *TP53* mutations, the mutation of *LRP1B* also implies a poor prognosis in older ovarian cancer patients. In this study, we detected a high frequency of *LRP1B* mutations in older ovarian cancer patients. Together, our results supported that *TP53*, *LRP1B*, and *BRCA1* were potential biomarkers for ovarian cancer patients. However, a shortcoming of this study was the small number of samples, and whether or not *FGFR1* and *LRP2* mutations specifically occurred in certain age groups still needs to be further confirmed with a larger patient cohort. We analyzed the mutations of *BRD4* and *STK11* from TCGA database and found that the mutation frequency of these two genes was (0.6%, 2/316)^21^. In this study, the mutation frequency of these two genes were 3% (2 / 65). Although the mutations of *BRD4* and *STK11* were associated with tumor stage in this study, the low frequency of *BRD4* and *STK11* mutations suggests that the sample population may be too small to cause false positive. Further studies with large population are needed to confirm this.

KRAS encodes a small GTPase and functions in many cellular processes by regulating its downstream pathways^35^. *KRAS* mutations have been considered as a biomarker for a more increased risk of ovarian cancer^36^, and its mutation status could also be a predictor for MEK inhibitor sensitivity in ovarian cancer^37^. *PTEN* is a tumor suppressor that regulates phosphatidylinositol 3-kinase (PI3K) signals^38^. Many studies have reported the importance of *PTEN* in ovarian cancer. Loss of *PTEN* may lead to a poor response to bortezomib in advanced ovarian cancer patients^39^, and the expression of *PTEN* was reported to be a prognosis biomarker in ovarian cancer^40^. Both *KRAS* and *PTEN* mutations commonly occurred in ovarian cancer^10^. Interestingly, even though only 4 cases with well/moderately differentiated tumors, we detected a correlation between *TP53*, *KRAS*, and *PTEN* mutations and tumor differentiation. This result supported that *TP53*, *KRAS*, and *PTEN* could be potential biomarkers as prognostic predictors of ovarian cancer. However, further confirmation is still needed. Tumor differentiation is associated with prognosis. Poorly differentiated tumors usually indicate a poor prognosis and well-differentiated tumors are more likely to indicate a good prognosis^41^. Su et al. reported that the high expression of miR-23a and the low expression of miR-23b were not only associated with medium/high differentiated tumors, but were also associated with the poor prognosis of ovarian cancer patients^42^. Combined with the correlation between the mutations of *TP53*, *KRAS*, and *PTEN* and tumor differentiation of ovarian cancer, it supports that tumor differentiation might be positively associated with prognosis in ovarian cancer.

Tumor heterogeneity is mainly due to the production of clones with metastatic potential and the existence of drug resistant mutations^43^. Metastasis is the main cause of malignant transformation and death for most cancer patients^44^. A large number of ovarian cancer patients develop widespread cancer cells beyond the ovaries or distant metastasis^45^. Biomarkers of biological targets that are associated with ovarian cancer metastasis have been widely researched. Zhao et al*.* found that STAT4 is a key regulator of ovarian cancer metastasis^46^. Wang et al*.* reported that the expression of *MTA1* was reported to be associated with metastasis of ovarian cancer^47^. Grither et al*.* reported that discoidin domain receptor 2 (DDR2), a receptor tyrosine kinase (RTK), is a potential target for the treatment of metastatic ovarian cancer^48^. In this study, although no significant correlation between mutated genes and tumor metastasis was detected, high mutational frequencies of *LRP2* and *NTRK3* were detected in metastatic tumors. *NTRK3* had been reported to be a prognosis predictor of ovarian cancer based on the correlation between *NTRK3* CNVs and platinum-sensitive and platinum-resistant recurrences^49^. Festuccia et al*.* also reported that CEP-701, a pan TRK inhibitor, could effectively reduce metastasis in advanced prostate cancer^50^. Recently, Tian et al*.* explained that *LRP2* played an important role in tumor cell motility and the tumor metastasis mechanism regulated by Hsp90α^51^. All these studies support our conclusion from this study that *NTRK3* and *LRP2* might be prognosis biomarkers for Chinese ovarian cancer patients.

Wang et al*.* investigated the molecular profiles and analyzed TMB in Chinese patients with gynecological cancers, including ovarian, cervical, and endometrial cancers, and found that the mutation of *BRCA1* was associated with higher TMB in ovarian cancer patients^52^. Birkbak et al*.* studied TMB in ovarian cancer with *BRCA1* and *BRCA2* and found that TMB coupled with *BRCA1* or *BRCA2* mutations could be used as a genomic marker of prognosis and a predictor of treatment response^53^. Although most *BRCA1/2* mutations occurred in the TMB-L group, we did not detect a correlation between *BRCA1/2* mutations and TMB in this study, which might be due to the small number of patients in this cohort. However, correlations between the mutations of *MED12*, *LRP2*, *PIK3R2*, *CCNE1*, and *LRP1B* and TMB-H were detected. TMB-H has been reported to correlate with the generation of neoantigens and potential clinical responses to immunotherapies in many cancer types^54,55^. A case report of a 71-year-old female with platinum-resistant ovarian cancer also showed that TMB might be a biomarker for immunotherapy^56^. Together, we deduced that *MED12*, *LRP2*, *PIK3R2*, *CCNE1*, and *LRP1B* might be potential biomarkers for immunotherapy of ovarian cancer.

Previous studies showed that 96% of ovarian cancer patients had *TP53* mutation, while the frequencies of other mutations were less than 10%^21^. In this study, in addition to the high frequency *TP53* mutations, we also identified a series of somatic mutations such as *NF1*, *NOTCH3*, *PRKCI*, *TERT, FAM135B*, *MYC*, *NOTCH1*, and *PTK2*, which were high frequently occurred and significantly higher in Chinese than those in Western patients, suggesting different mutational patterns of Chinese and Western patients. The high frequency mutations in this cohort means that Chinese ovarian cancer patients may share more common mutation, which is of great significance for the development of targeted treatment and precise treatment for further ovarian cancer treatment. The common germline mutations in ovarian cancer includes *BRCA1*, *BRCA2*, *ATM*, *MSH3* and *PALB2*^57^. The most common germline mutations are *BRCA1* and *BRCA2*^57^. In this study, we identified 25 germline mutations from 25 patients. Interestingly, the frequency of *BRCA1* germline mutations in Chinese is significantly higher than that in Western ovarian cancer patients, which also supported the different mutational patterns of ovarian cancer patients in different regions. Other germline mutations, such as *BRCA2*, *RAD51D*, *RAD51C*, and *FANCA*, were also identified in this study. They are all associated with homologous recombination deficiency (HRD)^58^, suggesting that nearly half of Chinese ovarian patients may benefit from Polyadenosine-diphosphate-ribose polymerase (PARP) inhibitors.

PARP inhibitors were considered to improve progression-free survival (PFS) of platinum-sensitive ovarian cancer patients^59,60^. In this study, we identified the mutations of six platinum sensitive patients who were received olaparib treatment. Interestingly, *BRCA1* rearrangement was found in an olaparib benefited patient. The *BRCA* mutations in response to PAPR inhibitors is complex. So far, few studies had reported that *BRCA1* rearrangement in ovarian cancer was responsive to olaparib. Our result suggested that patients with *BRCA* rearrangement might also be sensitive to olaparib. Meanwhile, we also found the amplification of *AURKA* and *RAD21* and SNV mutation of *AURKA* in 2 olaparib benefited patients. Both *AURKA* and *RAD21* were reported to relate with DNA repair system, and so that considered as HRD genes^61,62^. Many studies have shown that ovarian cancer patients with HRD related mutations were a target for PARP inhibitors^63^. However, both *BRCA* mutations and reported HRD related mutations failed to detected in two patients who benefits from olaparib in this study. This may be due to other mechanisms and further research is needed. Interestingly, we found deletions of *GATA4* and *LRP1B* in a patient who failed to response to olaparib. *GATA4* and *LRP1B* were tumor suppressor genes^32,64^, which might be related to the resistance of olaparib. However, only one patient tested is not enough to support this deduce, and more relevant cases still needed for further research.

In conclusion, we identified the genomic landscape of Chinese ovarian cancer patients and identified the correlation between mutated genes and clinical features including patients’ age, tumor differentiation, tumor lesion site, and TMB value. A series of potential biomarkers were identified for the prognosis of ovarian cancer patients. Our results supported that olaparib is effective in platinum sensitive patients with *BRCA* mutation and HRD related mutations. Although we had a limited number of samples, our study has enriched the understanding of the genomic mutational features of ovarian cancer and provides a basis for further development and application of molecular targeted therapy for ovarian cancer patients.

## Supplementary Information

Supplementary Information.

Supplementary Information.

Supplementary Information.

Supplementary Information.

Supplementary Information.

Supplementary Information.

Supplementary Information.

Supplementary Information.

Supplementary Information.

## Data Availability

The datasets used and analyzed in this study are available from the corresponding author upon reasonable request.
