# Supplementary material for: A comprehensive analysis of somatic alterations in Chinese ovarian cancer patients
Source: Sci Rep. 2021 Jan 11;11:387. doi: 10.1038/s41598-020-79694-0 (PMC7801677; doi:10.1038/s41598-020-79694-0)
Supplement: Supplementary file 1 — Supplementary Information. [file 41598_2020_79694_MOESM1_ESM.doc]

**The gene list of YuanSuTM450 gene panel.**

ABL1 ABL2 ACVR1B ACVR2A ADAM29 ADGRA2 AKT1 AKT2 AKT3 ALK AMER1 APC APEX1 AR ARAF ARFRP1 ARID1A ARID1B ARID2 ASXL1 ATF1 ATM ATR ATRX AURKA AURKB AXIN1 AXIN2 AXL BAP1 BARD1 BCL2 BCL2L1 BCL2L11 BCL2L2 BCL6 BCOR BCORL1 BCR BIRC5 BLK BLM BMPR1A BMX BRAF BRCA1 BRCA2 BRD4 BRIP1 BTG1 BTK CAMTA1 CARD11 CBFB CBL CCND1 CCND2 CCND3 CCNE1 CD274 CD79A CD79B CDC73 CDH1 CDK12 CDK4 CDK6 CDK8 CDKN1A CDKN1B CDKN2A CDKN2B CDKN2C CEBPA CFTR CHD2 CHD4 CHEK1 CHEK2 CIC COL1A1 CRBN CREB3L1 CREB3L2 CREBBP CRKL CRLF2 CSF1 CSF1R CSK CSNK1A1 CTCF CTNNA1 CTNNB1 CUL3 CXCR4 CYLD CYP17A1 CYP2D6 DAXX DDR1 DDR2 DICER1 DNMT3A DOT1L DPYD EGF EGFR EMSY EP300 EPCAM EPHA2 EPHA3 EPHA5 EPHA7 EPHB1 ERBB2 ERBB3 ERBB4 ERCC1 ERG ERRFI1 ESR1 ETV1 ETV4 ETV5 ETV6 EWSR1 EZH2 FAM135B TENT5C FANCA FANCC FANCD2 FANCE FANCF FANCG FANCL FANCM FAS FAT1 FAT3 FAT4 FBXW7 FEN1 FEV FGF10 FGF12 FGF14 FGF19 FGF23 FGF3 FGF4 FGF6 FGF7 FGFR1 FGFR2 FGFR3 FGFR4 FGR FH FLCN FLI1 FLT1 FLT3 FLT4 FOS FOXL2 FOXO1 FOXP1 FRS2 FUBP1 FUS FYN GABRA6 GATA1 GATA2 GATA3 GATA4 GATA6 GID4 GLI1 GLI2 GLI3 GNA11 GNA13 GNAQ GNAS GRIN2A GRM3 GSK3B H3-3A HCK HDAC9 HGF HNF1A HRAS HSD3B1 HSP90AA1 HTATIP2 IDH1 IDH2 IGF1R IGF2 IKBKE IKZF1 IL7R INHBA INPP4B IRF2 IRF4 IRS2 ITK JAK1 JAK2 JAK3 JUN KAT6A KDM5A KDM5B KDM5C KDM6A KDR KEAP1 KEL KIT KLHL6 KMT2A KMT2C KMT2D KRAS LCK LIMK1 LMO1 LRP1 LRP1B LRP2 LYN LZTR1 MACC1 MAGI2 MAP2K1 MAP2K2 MAP2K4 MAP3K1 MAP3K13 MAP4K5 MCL1 MDM2 MDM4 MED12 MEF2B MEN1 MERTK MET MGMT MITF MLH1 MPL MRE11 MS4A1 MSH2 MSH6 MST1R MTOR MUTYH MYB MYC MYCL MYCN MYD88 NBN NCOA2 NCOR1 NEK11 NF1 NF2 NFE2L2 NFIB NFKBIA NKX2-1 NOTCH1 NOTCH2 NOTCH3 NOTCH4 NPM1 NR4A3 NRAS NRG1 NRG3 NSD1 NTRK1 NTRK2 NTRK3 NUP93 PAK3 PALB2 PRKN PARP1 PARP2 PARP3 PARP4 PAX5 PBRM1 PCA3 PDCD1 PDCD1LG2 PDGFB PDGFRA PDGFRB PDK1 PIK3C2B PIK3CA PIK3CB PIK3CD PIK3CG PIK3R1 PIK3R2 PKD2 PLA2G1B PLCG2 PMS2 POLB POLD1 POLE PPP2R1A PRDM1 PREX2 PRKACA PRKAR1A PRKCI PRKDC PRSS1 PRSS8 PTCH1 PTEN PTK2 PTK6 PTPN11 QKI RAC1 RAD50 RAD51 RAD51B RAD51C RAD51D RAD52 RAD54B RAD54L RAF1 RANBP2 RARA RB1 RBM10 RECQL REL RELA RELB RET RHBDF2 RHOA RICTOR RNF43 ROCK1 ROCK2 ROS1 RPTOR RUNX1 RUNX1T1 RXRA SDHA SDHB SDHC SDHD SETD2 SF3B1 SIK1 SLIT2 SMAD2 SMAD3 SMAD4 SMARCA4 SMARCB1 SMARCD1 SMO SNCAIP SOCS1 SOX10 SOX2 SOX9 SPEN SPINK1 SPOP SPTA1 SRC SRMS SS18 SSX1 STAG2 STAT3 STAT4 STK11 STK24 SUFU SYK TAF1 TBX3 TCF7L2 TEK TERT TET1 TET2 TET3 TFE3 TGFBR1 TGFBR2 TIE1 TIPARP TMPRSS2 TNFAIP3 TNFRSF14 TNFSF11 TNFSF13B TNK2 TOP1 TOP2A TP53 TPMT TSC1 TSC2 TSHR TYK2 U2AF1 UGT1A1 VEGFA VHL WEE1 WEE2 NSD2 CCN6 WT1 XIAP XPO1 XRCC2 XRCC3 YES1 ZBTB2 ZNF217 ZNF703 ZNF750
