# Supplementary material for: A comprehensive analysis of somatic alterations in Chinese ovarian cancer patients
Source: Sci Rep. 2021 Jan 11;11:387. doi: 10.1038/s41598-020-79694-0 (PMC7801677; doi:10.1038/s41598-020-79694-0)
Supplement: Supplementary file 3 — Supplementary Information. [file 41598_2020_79694_MOESM3_ESM.docx]

Table S1 Total genomic alterations detected in different genes

| Genes | CNV | Fusion | LONGInDel | SNV | Mutation Frequency |
| --- | --- | --- | --- | --- | --- |
| *TP53* | 0 | 0 | 0 | 56 | 86.15% |
| *NF1* | 2 | 2 | 3 | 2 | 13.85% |
| *NOTCH3* | 2 | 0 | 1 | 4 | 10.77% |
| *TERT* | 6 | 0 | 0 | 1 | 10.77% |
| *BRCA1* | 0 | 2 | 3 | 1 | 9.23% |
| *MYC* | 5 | 0 | 0 | 1 | 9.23% |
| *PRKCI* | 6 | 0 | 0 | 0 | 9.23% |
| *ARID1A* | 0 | 2 | 0 | 3 | 7.69% |
| *EZH2* | 4 | 0 | 0 | 1 | 7.69% |
| *FAM135B* | 3 | 0 | 0 | 2 | 7.69% |
| *FGF12* | 4 | 0 | 0 | 1 | 7.69% |
| *FGFR3* | 4 | 0 | 0 | 1 | 7.69% |
| *GNAS* | 4 | 0 | 0 | 1 | 7.69% |
| *LRP1B* | 0 | 0 | 1 | 4 | 7.69% |
| *NOTCH1* | 3 | 0 | 0 | 2 | 7.69% |
| *PIK3CA* | 2 | 0 | 0 | 3 | 7.69% |
| *PTK2* | 5 | 0 | 0 | 0 | 7.69% |
| *SDHA* | 5 | 0 | 0 | 0 | 7.69% |
| *VEGFA* | 4 | 0 | 0 | 1 | 7.69% |
| *ATM* | 0 | 1 | 0 | 3 | 6.15% |
| *AURKA* | 3 | 0 | 0 | 1 | 6.15% |
| *CCND1* | 4 | 0 | 0 | 0 | 6.15% |
| *CCNE1* | 4 | 0 | 0 | 0 | 6.15% |
| *IGF1R* | 3 | 0 | 0 | 1 | 6.15% |
| *KMT2A* | 1 | 2 | 0 | 1 | 6.15% |
| *MECOM* | 4 | 0 | 0 | 0 | 6.15% |
| *MUC16* | 1 | 0 | 0 | 3 | 6.15% |
| *NSD2* | 4 | 0 | 0 | 0 | 6.15% |
| *PTEN* | 2 | 0 | 0 | 2 | 6.15% |
| *SRMS* | 3 | 0 | 0 | 1 | 6.15% |
| *TNK2* | 4 | 0 | 0 | 0 | 6.15% |
| *ARFRP1* | 3 | 0 | 0 | 0 | 4.62% |
| *BCL6* | 3 | 0 | 0 | 0 | 4.62% |
| *BCOR* | 1 | 0 | 0 | 2 | 4.62% |
| *BRCA2* | 0 | 2 | 0 | 1 | 4.62% |
| *CDK12* | 0 | 1 | 0 | 2 | 4.62% |
| *CFTR* | 2 | 0 | 0 | 1 | 4.62% |
| *FGF14* | 2 | 0 | 0 | 1 | 4.62% |
| *FGF19* | 2 | 0 | 0 | 1 | 4.62% |
| *FGFR1* | 2 | 0 | 0 | 1 | 4.62% |
| *GATA3* | 3 | 0 | 0 | 0 | 4.62% |
| *GRIN2A* | 1 | 0 | 0 | 2 | 4.62% |
| *ITK* | 2 | 0 | 0 | 1 | 4.62% |
| *KRAS* | 0 | 0 | 0 | 3 | 4.62% |
| *LRP2* | 0 | 0 | 0 | 3 | 4.62% |
| *MAP4K5* | 1 | 0 | 1 | 1 | 4.62% |
| *MET* | 2 | 0 | 0 | 1 | 4.62% |
| *NFKBIA* | 0 | 3 | 0 | 0 | 4.62% |
| *NTRK3* | 0 | 2 | 0 | 1 | 4.62% |
| *PIK3C2G* | 0 | 0 | 0 | 3 | 4.62% |
| *PIK3R2* | 1 | 0 | 0 | 2 | 4.62% |
| *PRKDC* | 1 | 1 | 0 | 1 | 4.62% |
| *PTK6* | 3 | 0 | 0 | 0 | 4.62% |
| *RAD52* | 3 | 0 | 0 | 0 | 4.62% |
| *RECQL4* | 3 | 0 | 0 | 0 | 4.62% |
| *TP63* | 3 | 0 | 0 | 0 | 4.62% |
| *TSC2* | 0 | 1 | 0 | 2 | 4.62% |
| *ZNF217* | 3 | 0 | 0 | 0 | 4.62% |
| *ARAF* | 2 | 0 | 0 | 0 | 3.08% |
| *BCL2L1* | 2 | 0 | 0 | 0 | 3.08% |
| *BRAF* | 2 | 0 | 0 | 0 | 3.08% |
| *BRD4* | 2 | 0 | 0 | 0 | 3.08% |
| *CHD4* | 1 | 0 | 0 | 1 | 3.08% |
| *CREB3L1* | 0 | 1 | 0 | 1 | 3.08% |
| *CREBBP* | 0 | 0 | 0 | 2 | 3.08% |
| *CRLF2* | 2 | 0 | 0 | 0 | 3.08% |
| *CUL4A* | 2 | 0 | 0 | 0 | 3.08% |
| *DDR1* | 1 | 1 | 0 | 0 | 3.08% |
| *EPHA3* | 0 | 0 | 0 | 2 | 3.08% |
| *ETV5* | 2 | 0 | 0 | 0 | 3.08% |
| *FANCC* | 0 | 2 | 0 | 0 | 3.08% |
| *FGF3* | 2 | 0 | 0 | 0 | 3.08% |
| *FGF4* | 2 | 0 | 0 | 0 | 3.08% |
| *FGFR2* | 1 | 0 | 0 | 1 | 3.08% |
| *FLI1* | 2 | 0 | 0 | 0 | 3.08% |
| *FUS* | 0 | 0 | 1 | 1 | 3.08% |
| *IRF4* | 2 | 0 | 0 | 0 | 3.08% |
| *JAK3* | 1 | 1 | 0 | 0 | 3.08% |
| *KDM5A* | 2 | 0 | 0 | 0 | 3.08% |
| *KDM6A* | 1 | 0 | 0 | 1 | 3.08% |
| *KIT* | 1 | 0 | 0 | 1 | 3.08% |
| *KMT2C* | 0 | 0 | 0 | 2 | 3.08% |
| *KMT2D* | 0 | 0 | 0 | 2 | 3.08% |
| *LRP1* | 0 | 0 | 0 | 2 | 3.08% |
| *LYN* | 2 | 0 | 0 | 0 | 3.08% |
| *LZTR1* | 1 | 0 | 0 | 1 | 3.08% |
| *MAP3K1* | 0 | 0 | 1 | 1 | 3.08% |
| *MCF2L* | 2 | 0 | 0 | 0 | 3.08% |
| *MED12* | 0 | 0 | 0 | 2 | 3.08% |
| *MEF2B* | 2 | 0 | 0 | 0 | 3.08% |
| *NSD1* | 1 | 0 | 0 | 1 | 3.08% |
| *PAK1* | 2 | 0 | 0 | 0 | 3.08% |
| *PPP2R2A* | 0 | 0 | 0 | 2 | 3.08% |
| *RB1* | 1 | 0 | 0 | 1 | 3.08% |
| *RBM10* | 0 | 1 | 0 | 1 | 3.08% |
| *RET* | 1 | 0 | 0 | 1 | 3.08% |
| *RICTOR* | 1 | 0 | 0 | 1 | 3.08% |
| *RPTOR* | 1 | 0 | 0 | 1 | 3.08% |
| *RSPO2* | 1 | 0 | 0 | 1 | 3.08% |
| *RUNX1* | 0 | 0 | 1 | 1 | 3.08% |
| *RXRA* | 2 | 0 | 0 | 0 | 3.08% |
| *SMAD4* | 0 | 0 | 0 | 2 | 3.08% |
| *SRC* | 2 | 0 | 0 | 0 | 3.08% |
| *STK11* | 1 | 1 | 0 | 0 | 3.08% |
| *STK24* | 2 | 0 | 0 | 0 | 3.08% |
| *TAF1* | 0 | 0 | 0 | 2 | 3.08% |
| *TERC* | 2 | 0 | 0 | 0 | 3.08% |
| *TRIO* | 2 | 0 | 0 | 0 | 3.08% |
| *ABCB1* | 0 | 0 | 0 | 1 | 1.54% |
| *ACVR2A* | 1 | 0 | 0 | 0 | 1.54% |
| *ADGRA2* | 1 | 0 | 0 | 0 | 1.54% |
| *AGRN* | 0 | 0 | 0 | 1 | 1.54% |
| *AKT1* | 1 | 0 | 0 | 0 | 1.54% |
| *AMER1* | 0 | 0 | 0 | 1 | 1.54% |
| *ANKRD36* | 0 | 0 | 0 | 1 | 1.54% |
| *APC* | 0 | 0 | 0 | 1 | 1.54% |
| *APEX1* | 1 | 0 | 0 | 0 | 1.54% |
| *APLNR* | 0 | 0 | 0 | 1 | 1.54% |
| *ARHGAP6* | 0 | 0 | 0 | 1 | 1.54% |
| *ARHGEF17* | 1 | 0 | 0 | 0 | 1.54% |
| *ARID1B* | 0 | 1 | 0 | 0 | 1.54% |
| *ASXL1* | 1 | 0 | 0 | 0 | 1.54% |
| *ATP1A2* | 0 | 0 | 0 | 1 | 1.54% |
| *ATR* | 0 | 0 | 0 | 1 | 1.54% |
| *ATRX* | 0 | 0 | 0 | 1 | 1.54% |
| *BCHE* | 0 | 0 | 0 | 1 | 1.54% |
| *BCL2L11* | 0 | 1 | 0 | 0 | 1.54% |
| *BCR* | 1 | 0 | 0 | 0 | 1.54% |
| *BIRC3* | 0 | 0 | 0 | 1 | 1.54% |
| *BIRC5* | 1 | 0 | 0 | 0 | 1.54% |
| *BMX* | 0 | 0 | 0 | 1 | 1.54% |
| *BTK* | 1 | 0 | 0 | 0 | 1.54% |
| *C19orf12* | 0 | 0 | 0 | 1 | 1.54% |
| *C9orf129* | 0 | 0 | 0 | 1 | 1.54% |
| *CACNA1A* | 0 | 0 | 0 | 1 | 1.54% |
| *CACNA1C* | 0 | 0 | 0 | 1 | 1.54% |
| *CARD11* | 1 | 0 | 0 | 0 | 1.54% |
| *CCDC88A* | 0 | 0 | 0 | 1 | 1.54% |
| *CCND2* | 1 | 0 | 0 | 0 | 1.54% |
| *CCND3* | 1 | 0 | 0 | 0 | 1.54% |
| *CD70* | 1 | 0 | 0 | 0 | 1.54% |
| *CDC20B* | 0 | 0 | 0 | 1 | 1.54% |
| *CDK8* | 0 | 0 | 0 | 1 | 1.54% |
| *CDKN1B* | 0 | 0 | 0 | 1 | 1.54% |
| *CDKN2A* | 1 | 0 | 0 | 0 | 1.54% |
| *CDKN2B* | 1 | 0 | 0 | 0 | 1.54% |
| *CIC* | 0 | 0 | 0 | 1 | 1.54% |
| *CKM* | 0 | 0 | 0 | 1 | 1.54% |
| *CLCN5* | 0 | 0 | 0 | 1 | 1.54% |
| *CNGA4* | 0 | 0 | 0 | 1 | 1.54% |
| *COL1A1* | 1 | 0 | 0 | 0 | 1.54% |
| *CPS1* | 0 | 0 | 0 | 1 | 1.54% |
| *CREB3L2* | 1 | 0 | 0 | 0 | 1.54% |
| *CRKL* | 1 | 0 | 0 | 0 | 1.54% |
| *CSK* | 1 | 0 | 0 | 0 | 1.54% |
| *CSMD3* | 0 | 0 | 0 | 1 | 1.54% |
| *CTNNB1* | 1 | 0 | 0 | 0 | 1.54% |
| *CYP2D6* | 1 | 0 | 0 | 0 | 1.54% |
| *DHX35* | 0 | 0 | 0 | 1 | 1.54% |
| *DHX37* | 0 | 0 | 0 | 1 | 1.54% |
| *DICER1* | 0 | 0 | 0 | 1 | 1.54% |
| *DNMT1* | 0 | 0 | 0 | 1 | 1.54% |
| *DNMT3B* | 0 | 0 | 0 | 1 | 1.54% |
| *ECT2* | 1 | 0 | 0 | 0 | 1.54% |
| *EED* | 1 | 0 | 0 | 0 | 1.54% |
| *EGFR* | 0 | 0 | 0 | 1 | 1.54% |
| *EMSY* | 1 | 0 | 0 | 0 | 1.54% |
| *EP300* | 0 | 0 | 0 | 1 | 1.54% |
| *EPHA2* | 1 | 0 | 0 | 0 | 1.54% |
| *EPHA5* | 0 | 0 | 0 | 1 | 1.54% |
| *EPHB4* | 1 | 0 | 0 | 0 | 1.54% |
| *ERBB2* | 0 | 0 | 0 | 1 | 1.54% |
| *ERBB4* | 0 | 0 | 0 | 1 | 1.54% |
| *ERCC5* | 1 | 0 | 0 | 0 | 1.54% |
| *ETV1* | 0 | 0 | 0 | 1 | 1.54% |
| *ETV6* | 0 | 0 | 0 | 1 | 1.54% |
| *EZR* | 1 | 0 | 0 | 0 | 1.54% |
| *FAM120B* | 0 | 0 | 0 | 1 | 1.54% |
| *FAS* | 1 | 0 | 0 | 0 | 1.54% |
| *FAT1* | 0 | 0 | 0 | 1 | 1.54% |
| *FAT3* | 0 | 0 | 0 | 1 | 1.54% |
| *FGF18* | 1 | 0 | 0 | 0 | 1.54% |
| *FGF23* | 1 | 0 | 0 | 0 | 1.54% |
| *FGF5* | 0 | 0 | 0 | 1 | 1.54% |
| *FGF6* | 1 | 0 | 0 | 0 | 1.54% |
| *FGFR4* | 1 | 0 | 0 | 0 | 1.54% |
| *FGR* | 1 | 0 | 0 | 0 | 1.54% |
| *FLT4* | 1 | 0 | 0 | 0 | 1.54% |
| *FSHR* | 0 | 0 | 0 | 1 | 1.54% |
| *GAL3ST2* | 0 | 0 | 0 | 1 | 1.54% |
| *GATA4* | 1 | 0 | 0 | 0 | 1.54% |
| *GATA6* | 0 | 0 | 0 | 1 | 1.54% |
| *GIF* | 0 | 0 | 0 | 1 | 1.54% |
| *GLI2* | 1 | 0 | 0 | 0 | 1.54% |
| *GLI3* | 0 | 0 | 0 | 1 | 1.54% |
| *HCK* | 1 | 0 | 0 | 0 | 1.54% |
| *HDAC1* | 1 | 0 | 0 | 0 | 1.54% |
| *HSP90AA1* | 1 | 0 | 0 | 0 | 1.54% |
| *IDH2* | 1 | 0 | 0 | 0 | 1.54% |
| *IKBKE* | 1 | 0 | 0 | 0 | 1.54% |
| *IL7R* | 0 | 0 | 0 | 1 | 1.54% |
| *KAT6A* | 1 | 0 | 0 | 0 | 1.54% |
| *KDM5B* | 1 | 0 | 0 | 0 | 1.54% |
| *KDR* | 1 | 0 | 0 | 0 | 1.54% |
| *KEL* | 0 | 0 | 0 | 1 | 1.54% |
| *KLHL6* | 1 | 0 | 0 | 0 | 1.54% |
| *LAMA3* | 0 | 0 | 0 | 1 | 1.54% |
| *LOX* | 0 | 0 | 0 | 1 | 1.54% |
| *MAGEB6* | 0 | 0 | 0 | 1 | 1.54% |
| *MAGI2* | 0 | 0 | 0 | 1 | 1.54% |
| *MAP2K2* | 1 | 0 | 0 | 0 | 1.54% |
| *MAP2K4* | 0 | 0 | 0 | 1 | 1.54% |
| *MAP3K13* | 1 | 0 | 0 | 0 | 1.54% |
| *MAPK1* | 1 | 0 | 0 | 0 | 1.54% |
| *MBD2* | 0 | 0 | 0 | 1 | 1.54% |
| *MC2R* | 0 | 0 | 0 | 1 | 1.54% |
| *MCM6* | 0 | 0 | 0 | 1 | 1.54% |
| *MDM4* | 1 | 0 | 0 | 0 | 1.54% |
| *MLH1* | 0 | 0 | 0 | 1 | 1.54% |
| *MST1R* | 0 | 0 | 0 | 1 | 1.54% |
| *MYH11* | 0 | 0 | 0 | 1 | 1.54% |
| *NAA20* | 0 | 0 | 0 | 1 | 1.54% |
| *NCKIPSD* | 0 | 0 | 0 | 1 | 1.54% |
| *NCOA2* | 0 | 0 | 0 | 1 | 1.54% |
| *NDST2* | 0 | 0 | 0 | 1 | 1.54% |
| *NID2* | 0 | 0 | 0 | 1 | 1.54% |
| *NPY1R* | 0 | 0 | 0 | 1 | 1.54% |
| *NRG1* | 0 | 0 | 0 | 1 | 1.54% |
| *NRG3* | 0 | 0 | 0 | 1 | 1.54% |
| *OBSCN* | 0 | 0 | 0 | 1 | 1.54% |
| *OIT3* | 0 | 0 | 0 | 1 | 1.54% |
| *OR4K17* | 0 | 0 | 0 | 1 | 1.54% |
| *OR9G4* | 0 | 0 | 0 | 1 | 1.54% |
| *PAGE2* | 0 | 0 | 0 | 1 | 1.54% |
| *PARK2* | 1 | 0 | 0 | 0 | 1.54% |
| *PARP2* | 0 | 1 | 0 | 0 | 1.54% |
| *PARP4* | 0 | 0 | 0 | 1 | 1.54% |
| *PAX5* | 0 | 0 | 0 | 1 | 1.54% |
| *PDK1* | 1 | 0 | 0 | 0 | 1.54% |
| *PIK3C2B* | 1 | 0 | 0 | 0 | 1.54% |
| *PIK3CB* | 1 | 0 | 0 | 0 | 1.54% |
| *PIK3CD* | 1 | 0 | 0 | 0 | 1.54% |
| *PIK3R1* | 0 | 0 | 0 | 1 | 1.54% |
| *PKN1* | 1 | 0 | 0 | 0 | 1.54% |
| *PLCD4* | 0 | 0 | 0 | 1 | 1.54% |
| *PLCG2* | 0 | 0 | 0 | 1 | 1.54% |
| *PML* | 1 | 0 | 0 | 0 | 1.54% |
| *POLB* | 1 | 0 | 0 | 0 | 1.54% |
| *POLE* | 0 | 0 | 0 | 1 | 1.54% |
| *POU1F1* | 0 | 0 | 0 | 1 | 1.54% |
| *PPP2R1A* | 0 | 0 | 0 | 1 | 1.54% |
| *PPP6R1* | 0 | 0 | 0 | 1 | 1.54% |
| *PRDM1* | 0 | 0 | 0 | 1 | 1.54% |
| *PRKACA* | 1 | 0 | 0 | 0 | 1.54% |
| *PRKAR1A* | 0 | 0 | 1 | 0 | 1.54% |
| *PRPS1* | 0 | 0 | 0 | 1 | 1.54% |
| *PTCH1* | 0 | 0 | 0 | 1 | 1.54% |
| *QKI* | 1 | 0 | 0 | 0 | 1.54% |
| *RAB4B* | 0 | 0 | 0 | 1 | 1.54% |
| *RAD21* | 1 | 0 | 0 | 0 | 1.54% |
| *RAD51* | 0 | 0 | 0 | 1 | 1.54% |
| *RAD51D* | 0 | 1 | 0 | 0 | 1.54% |
| *RAD54L* | 1 | 0 | 0 | 0 | 1.54% |
| *RASGRF1* | 0 | 0 | 0 | 1 | 1.54% |
| *RASGRP3* | 0 | 0 | 0 | 1 | 1.54% |
| *REL* | 0 | 0 | 0 | 1 | 1.54% |
| *RELA* | 1 | 0 | 0 | 0 | 1.54% |
| *RHBDF2* | 1 | 0 | 0 | 0 | 1.54% |
| *RINL* | 0 | 0 | 0 | 1 | 1.54% |
| *RIT1* | 1 | 0 | 0 | 0 | 1.54% |
| *RORB* | 0 | 0 | 0 | 1 | 1.54% |
| *RPGRIP1L* | 0 | 0 | 0 | 1 | 1.54% |
| *RPS24* | 0 | 0 | 0 | 1 | 1.54% |
| *SETD2* | 0 | 0 | 0 | 1 | 1.54% |
| *SLC15A2* | 0 | 0 | 0 | 1 | 1.54% |
| *SLC25A41* | 0 | 0 | 0 | 1 | 1.54% |
| *SLC26A7* | 0 | 0 | 0 | 1 | 1.54% |
| *SLC35B3* | 0 | 0 | 0 | 1 | 1.54% |
| *SLC6A19* | 0 | 0 | 0 | 1 | 1.54% |
| *SLC6A8* | 0 | 0 | 0 | 1 | 1.54% |
| *SLIT2* | 0 | 0 | 1 | 0 | 1.54% |
| *SMAD3* | 0 | 0 | 0 | 1 | 1.54% |
| *SMARCA4* | 0 | 1 | 0 | 0 | 1.54% |
| *SMARCB1* | 0 | 0 | 0 | 1 | 1.54% |
| *SOX7* | 0 | 0 | 0 | 1 | 1.54% |
| *SPTA1* | 0 | 0 | 1 | 0 | 1.54% |
| *SRGAP3* | 0 | 0 | 0 | 1 | 1.54% |
| *STAG2* | 0 | 0 | 0 | 1 | 1.54% |
| *SYT14* | 0 | 0 | 0 | 1 | 1.54% |
| *TCF7L2* | 1 | 0 | 0 | 0 | 1.54% |
| *TEK* | 0 | 0 | 0 | 1 | 1.54% |
| *TET1* | 0 | 0 | 0 | 1 | 1.54% |
| *TET3* | 0 | 0 | 1 | 0 | 1.54% |
| *TFAP4* | 0 | 0 | 0 | 1 | 1.54% |
| *TFEB* | 1 | 0 | 0 | 0 | 1.54% |
| *TGFBR1* | 0 | 0 | 0 | 1 | 1.54% |
| *TIPARP* | 1 | 0 | 0 | 0 | 1.54% |
| *TMED2* | 0 | 0 | 0 | 1 | 1.54% |
| *TNFSF13B* | 1 | 0 | 0 | 0 | 1.54% |
| *TNRC18* | 0 | 0 | 0 | 1 | 1.54% |
| *TSC22D2* | 0 | 0 | 0 | 1 | 1.54% |
| *TSPAN1* | 1 | 0 | 0 | 0 | 1.54% |
| *TYK2* | 1 | 0 | 0 | 0 | 1.54% |
| *UBQLN2* | 0 | 0 | 0 | 1 | 1.54% |
| *UNC13C* | 0 | 0 | 0 | 1 | 1.54% |
| *UROC1* | 0 | 0 | 0 | 1 | 1.54% |
| *USH2A* | 0 | 0 | 0 | 1 | 1.54% |
| *VLDLR* | 0 | 0 | 0 | 1 | 1.54% |
| *VWCE* | 0 | 0 | 0 | 1 | 1.54% |
| *VWF* | 0 | 0 | 0 | 1 | 1.54% |
| *WDFY4* | 0 | 0 | 0 | 1 | 1.54% |
| *WEE2* | 1 | 0 | 0 | 0 | 1.54% |
| *WT1* | 1 | 0 | 0 | 0 | 1.54% |
| *ZDHHC8* | 0 | 0 | 0 | 1 | 1.54% |
| *ZIK1* | 0 | 0 | 0 | 1 | 1.54% |
| *ZNF703* | 1 | 0 | 0 | 0 | 1.54% |
| *ZNF714* | 0 | 0 | 0 | 1 | 1.54% |
