# Supplementary material for: A comprehensive analysis of somatic alterations in Chinese ovarian cancer patients
Source: Sci Rep. 2021 Jan 11;11:387. doi: 10.1038/s41598-020-79694-0 (PMC7801677; doi:10.1038/s41598-020-79694-0)
Supplement: Supplementary file 4 — Supplementary Information. [file 41598_2020_79694_MOESM4_ESM.docx]

Table S2 Germline mutations detected in this cohort

| Gene | Variation type | | Total |
| --- | --- | --- | --- |
|  | Substitution/Indel | Truncation |  |
| BRCA1 | 4 | 13 | 17 |
| BRCA2 | 1 | 2 | 3 |
| FANCA | 0 | 0 | 1 |
| RAD51C | 0 | 0 | 1 |
| RAD51D | 0 | 0 | 3 |
| Total |  |  | 25 |
