# Supplementary material for: A comprehensive analysis of somatic alterations in Chinese ovarian cancer patients
Source: Sci Rep. 2021 Jan 11;11:387. doi: 10.1038/s41598-020-79694-0 (PMC7801677; doi:10.1038/s41598-020-79694-0)
Supplement: Supplementary file 5 — Supplementary Information. [file 41598_2020_79694_MOESM5_ESM.docx]

Table S3 The most common genomic alterations in patients at different ages

| 33-49 | | | 50-59 | | | 60-84 | | |
| --- | --- | --- | --- | --- | --- | --- | --- | --- |
| Genes | Mutant Number | Mutation Frequency | Genes | Mutant Number | Mutation Frequency | Genes | Mutant Number | Mutation Frequency |
| TP53 | 14 | 77.78% | TP53 | 20 | 80.00% | TP53 | 22 | 100.00% |
| FGFR3 | 3 | 16.67% | NF1 | 6 | 24.00% | LRP1B | 4 | 18.18% |
| MYC | 3 | 16.67% | BRCA1 | 5 | 20.00% | CCNE1 | 3 | 13.64% |
| NSD2 | 3 | 16.67% | NOTCH3 | 5 | 20.00% | LRP2 | 3 | 13.64% |
| ARID1A | 2 | 11.11% | EZH2 | 3 | 12.00% | NOTCH1 | 3 | 13.64% |
| AURKA | 2 | 11.11% | FGFR1 | 3 | 12.00% |  |  |  |
| FAM135B | 2 | 11.11% | MYC | 3 | 12.00% |  |  |  |
| FGF12 | 2 | 11.11% | PRKCI | 3 | 12.00% |  |  |  |
| FGFR2 | 2 | 11.11% | SDHA | 3 | 12.00% |  |  |  |
| FLI1 | 2 | 11.11% | TERT | 3 | 12.00% |  |  |  |
| GNAS | 2 | 11.11% | VEGFA | 3 | 12.00% |  |  |  |
| KMT2A | 2 | 11.11% |  |  |  |  |  |  |
| KRAS | 2 | 11.11% |  |  |  |  |  |  |
| PIK3CA | 2 | 11.11% |  |  |  |  |  |  |
| PRKCI | 2 | 11.11% |  |  |  |  |  |  |
| PTK2 | 2 | 11.11% |  |  |  |  |  |  |
| SRMS | 2 | 11.11% |  |  |  |  |  |  |
| TERT | 2 | 11.11% |  |  |  |  |  |  |
| TNK2 | 2 | 11.11% |  |  |  |  |  |  |
| TSC2 | 2 | 11.11% |  |  |  |  |  |  |
