# Supplementary material for: A comprehensive analysis of somatic alterations in Chinese ovarian cancer patients
Source: Sci Rep. 2021 Jan 11;11:387. doi: 10.1038/s41598-020-79694-0 (PMC7801677; doi:10.1038/s41598-020-79694-0)
Supplement: Supplementary file 6 — Supplementary Information. [file 41598_2020_79694_MOESM6_ESM.docx]

Table S4 Correlation analysis between mutated genes and tumor differentiation.

| Genes | Poorly/undifferentiated | | Well/moderately | | P-value |
| --- | --- | --- | --- | --- | --- |
|  | Mutant Number | Mutation frequency | Mutant Number | Mutation frequency |  |
| KRAS | 0 | 0.00% | 3 | 75% | 0.000137 |
| PTEN | 1 | 1.89% | 2 | 50% | 0.011005 |
| TP53 | 50 | 94.33% | 2 | 50% | 0.034898 |
| NF1 | 7 | 13.21% | 1 | 25.00% | 0.463619 |
| NOTCH3 | 6 | 11.32% | 1 | 25.00% | 0.416977 |
| PRKCI | 6 | 11.32% | 0 | 0.00% | 0.893885 |
| MYC | 5 | 9.43% | 1 | 25.00% | 0.367358 |
| TERT | 5 | 9.43% | 1 | 25.00% | 0.367358 |
| BRCA1 | 5 | 9.43% | 0 | 0.00% | 0.784593 |
| EZH2 | 5 | 9.43% | 0 | 0.00% | 0.784593 |
| FAM135B | 5 | 9.43% | 0 | 0.00% | 0.784593 |
| LRP1B | 5 | 9.43% | 0 | 0.00% | 0.784593 |
| NOTCH1 | 5 | 9.43% | 0 | 0.00% | 0.784593 |
| PTK2 | 5 | 9.43% | 0 | 0.00% | 0.784593 |
