# Supplementary material for: A comprehensive analysis of somatic alterations in Chinese ovarian cancer patients
Source: Sci Rep. 2021 Jan 11;11:387. doi: 10.1038/s41598-020-79694-0 (PMC7801677; doi:10.1038/s41598-020-79694-0)
Supplement: Supplementary file 7 — Supplementary Information. [file 41598_2020_79694_MOESM7_ESM.docx]

Table S5 Correlation analysis between mutated genes and TMB value.

| Gene | TMB-H | | TMB-L | | P-value |
| --- | --- | --- | --- | --- | --- |
|  | Mutated Number | Mutation Frequency | Mutated Number | Mutation Frequency |  |
| TP53 | 3 | 75.00% | 51 | 94.44% | 0.361904 |
| NF1 | 1 | 25.00% | 10 | 18.52% | 0.50061 |
| TERT | 0 | 0.00% | 8 | 14.81% | 1 |
| MYC | 0 | 0.00% | 7 | 12.96% | 1 |
| FAM135B | 0 | 0.00% | 6 | 11.11% | 1 |
| PRKCI | 0 | 0.00% | 6 | 11.11% | 1 |
| NOTCH3 | 2 | 50.00% | 5 | 9.26% | 0.067398 |
| LRP1B | 3 | 75.00% | 3 | 5.56% | 0.03374 |
| PIK3R2 | 3 | 75.00% | 1 | 1.85% | 0.01063 |
| OBSCN | 3 | 75.00% | 0 | 0.00% | 0.068966 |
| CCNE1 | 2 | 50.00% | 2 | 3.70% | 0.020749 |
| LRP2 | 2 | 50.00% | 1 | 1.85% | 0.01063 |
| MED12 | 2 | 50.00% | 0 | 0.00% | 0.00363 |
