# Supplementary material for: A comprehensive analysis of somatic alterations in Chinese ovarian cancer patients
Source: Sci Rep. 2021 Jan 11;11:387. doi: 10.1038/s41598-020-79694-0 (PMC7801677; doi:10.1038/s41598-020-79694-0)
Supplement: Supplementary file 8 — Supplementary Information. [file 41598_2020_79694_MOESM8_ESM.docx]

Table S6 Genomic alterations in 6 platinum sensitive patients.

| Case ID | Mutated Genes | VAR_TYPE |
| --- | --- | --- |
| Case 1 | CNGA4 | SNV |
| Case 1 | TP53 | SNV |
| Case 1 | RINL | SNV |
| Case 1 | UROC1 | SNV |
| Case 1 | CDC20B | SNV |
| Case 1 | LOX | SNV |
| Case 1 | UBQLN2 | SNV |
| Case 1 | ITK | Gene Amplification |
| Case 1 | IRF4 | Gene Amplification |
| Case 1 | RSPO2 | Gene Amplification |
| Case 1 | RAD21 | Gene Amplification |
| Case 1 | MYC | Gene Amplification |
| Case 1 | FAM135B | Gene Amplification |
| Case 1 | PTK2 | Gene Amplification |
| Case 1 | RECQL4 | Gene Amplification |
| Case 1 | CCND1 | Gene Amplification |
| Case 1 | FGF19 | Gene Amplification |
| Case 1 | FGF4 | Gene Amplification |
| Case 1 | FGF3 | Gene Amplification |
| Case 1 | PAK1 | Gene Amplification |
| Case 1 | EED | Gene Amplification |
| Case 1 | CSK | Gene Deletion |
| Case 1 | CD70 | Gene Amplification |
| Case 1 | TYK2 | Gene Amplification |
| Case 1 | NOTCH3 | Gene Amplification |
| Case 1 | JAK3 | Gene Amplification |
| Case 1 | PIK3R2 | Gene Amplification |
| Case 1 | ZNF217 | Gene Amplification |
| Case 1 | AURKA | Gene Amplification |
| Case 2 | NDST2 | SNV |
| Case 2 | VWCE | SNV |
| Case 2 | NID2 | SNV |
| Case 2 | RASGRF1 | SNV |
| Case 2 | TFAP4 | SNV |
| Case 2 | TP53 | SNV |
| Case 2 | MBD2 | SNV |
| Case 2 | NOTCH3 | SNV |
| Case 2 | RAB4B | SNV |
| Case 2 | CKM | SNV |
| Case 2 | RASGRP3 | SNV |
| Case 2 | CPS1 | SNV |
| Case 2 | GAL3ST2 | SNV |
| Case 2 | NAA20 | SNV |
| Case 2 | FAM120B | SNV |
| Case 2 | SLC26A7 | SNV |
| Case 2 | CSMD3 | SNV |
| Case 2 | RORB | SNV |
| Case 2 | PAGE2 | SNV |
| Case 2 | TERT | Gene Amplification |
| Case 2 | CARD11 | Gene Amplification |
| Case 2 | BRCA1 | Rearrangement |
| Case 3 | AGRN | SNV |
| Case 3 | USH2A | SNV |
| Case 3 | OR9G4 | SNV |
| Case 3 | APLNR | SNV |
| Case 3 | TP53 | SNV |
| Case 3 | MC2R | SNV |
| Case 3 | LAMA3 | SNV |
| Case 3 | DNMT1 | SNV |
| Case 3 | CACNA1A | SNV |
| Case 3 | REL | SNV |
| Case 3 | AURKA | SNV |
| Case 3 | NCKIPSD | SNV |
| Case 3 | NPY1R | SNV |
| Case 3 | TNRC18 | SNV |
| Case 3 | C9orf129 | SNV |
| Case 3 | TNK2 | Gene Amplification |
| Case 3 | RECQL4 | Gene Amplification |
| Case 4 | ATP1A2 | SNV |
| Case 4 | UNC13C | SNV |
| Case 4 | UNC13C | SNV |
| Case 4 | TP53 | SNV |
| Case 4 | SLC25A41 | SNV |
| Case 4 | CCDC88A | SNV |
| Case 4 | CCDC88A | SNV |
| Case 4 | PLCD4 | SNV |
| Case 4 | TSC22D2 | SNV |
| Case 4 | KEL | SNV |
| Case 4 | SOX7 | SNV |
| Case 4 | VLDLR | SNV |
| Case 4 | MAGEB6 | SNV |
| Case 4 | CLCN5 | SNV |
| Case 4 | SLC6A8 | SNV |
| Case 5 | SYT14 | SNV |
| Case 5 | WDFY4 | SNV |
| Case 5 | DHX37 | SNV |
| Case 5 | RPGRIP1L | SNV |
| Case 5 | TP53 | SNV |
| Case 5 | PPP6R1 | SNV |
| Case 5 | DHX35 | SNV |
| Case 5 | SRMS | SNV |
| Case 5 | ZDHHC8 | SNV |
| Case 5 | SRGAP3 | SNV |
| Case 5 | SLC6A19 | SNV |
| Case 5 | SLC35B3 | SNV |
| Case 5 | PTCH1 | SNV |
| Case 5 | GRIN2A | CNV |
| Case 5 | STK11 | Rearrangement |
| Case 6 | OIT3 | SNV |
| Case 6 | RPS24 | SNV |
| Case 6 | GIF | SNV |
| Case 6 | CACNA1C | SNV |
| Case 6 | VWF | SNV |
| Case 6 | TMED2 | SNV |
| Case 6 | OR4K17 | SNV |
| Case 6 | TP53 | SNV |
| Case 6 | ZNF714 | SNV |
| Case 6 | C19orf12 | SNV |
| Case 6 | ZIK1 | SNV |
| Case 6 | FSHR | SNV |
| Case 6 | ANKRD36 | SNV |
| Case 6 | MCM6 | SNV |
| Case 6 | POU1F1 | SNV |
| Case 6 | SLC15A2 | SNV |
| Case 6 | BCHE | SNV |
| Case 6 | AMER1 | SNV |
| Case 6 | PRPS1 | SNV |
| Case 6 | ITK | Gene Amplification |
| Case 6 | PARK2 | Gene Homozygous Deletion |
| Case 6 | QKI | Gene Homozygous Deletion |
| Case 6 | GATA4 | Gene Homozygous Deletion |
| Case 6 | FGFR2 | Gene Amplification |
| Case 6 | PML | Gene Homozygous Deletion |
| Case 6 | ARAF | Gene Amplification |
| Case 6 | PRKAR1A | Gene Homozygous Deletion |
| Case 6 | LRP1B | Gene Homozygous Deletion |
