# Supplementary material for: A comprehensive analysis of somatic alterations in Chinese ovarian cancer patients
Source: Sci Rep. 2021 Jan 11;11:387. doi: 10.1038/s41598-020-79694-0 (PMC7801677; doi:10.1038/s41598-020-79694-0)
Supplement: Supplementary file 9 — Supplementary Information. [file 41598_2020_79694_MOESM9_ESM.pdf]

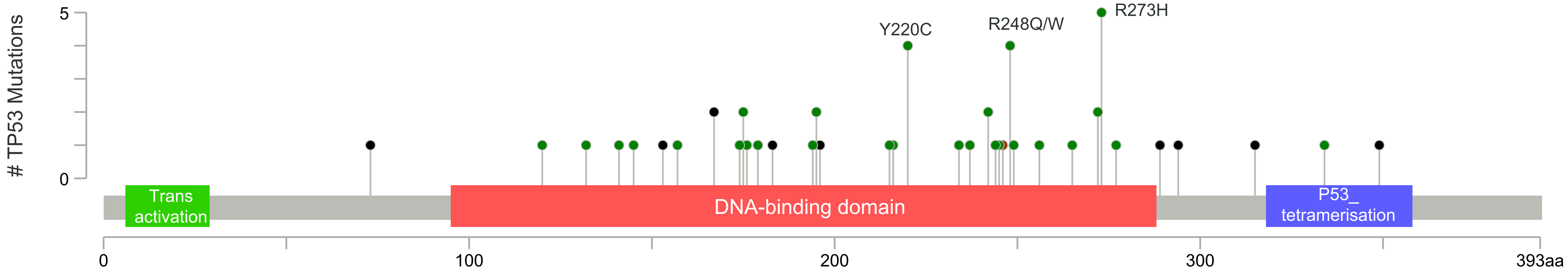

Figure S1 Distribution of TP53 genomic alterations. The X-axis shows the gene structure and the Y-axis shows the number of mutations in each site. Green represents the transactivation domain, red represents the DNA-binding domain, and blue represents the P53 tetramerization domain.
